# The Hinge Segment of Human NADPH-Cytochrome P450 Reductase in Conformational Switching: The Critical Role of Ionic Strength

**DOI:** 10.3389/fphar.2017.00755

**Published:** 2017-10-30

**Authors:** Diana Campelo, Thomas Lautier, Philippe Urban, Francisco Esteves, Sophie Bozonnet, Gilles Truan, Michel Kranendonk

**Affiliations:** ^1^Center for Toxicogenomics and Human Health (ToxOmics), Genetics, Oncology and Human Toxicology, NOVA Medical School, Faculdade de Ciências Médicas, Universidade Nova de Lisboa, Lisboa, Portugal; ^2^LISBP, Université de Toulouse, CNRS, INRA, INSA, Toulouse, France

**Keywords:** diflavin reductase, protein dynamics, multidomain proteins, conformational exchange, electron transfer, protein–protein interaction

## Abstract

NADPH-cytochrome P450 reductase (CPR) is a redox partner of microsomal cytochromes P450 and is a prototype of the diflavin reductase family. CPR contains 3 distinct functional domains: a FMN-binding domain (acceptor reduction), a linker (hinge), and a connecting/FAD domain (NADPH oxidation). It has been demonstrated that the mechanism of CPR exhibits an important step in which it switches from a compact, closed conformation (locked state) to an ensemble of open conformations (unlocked state), the latter enabling electron transfer to redox partners. The conformational equilibrium between the locked and unlocked states has been shown to be highly dependent on ionic strength, reinforcing the hypothesis of the presence of critical salt interactions at the interface between the FMN and connecting FAD domains. Here we show that specific residues of the hinge segment are important in the control of the conformational equilibrium of CPR. We constructed six single mutants and two double mutants of the human CPR, targeting residues G240, S243, I245 and R246 of the hinge segment, with the aim of modifying the flexibility or the potential ionic interactions of the hinge segment. We measured the reduction of cytochrome *c* at various salt concentrations of these 8 mutants, either in the soluble or membrane-bound form of human CPR. All mutants were found capable of reducing cytochrome *c* yet with different efficiency and their maximal rates of cytochrome *c* reduction were shifted to lower salt concentration. In particular, residue R246 seems to play a key role in a salt bridge network present at the interface of the hinge and the connecting domain. Interestingly, the effects of mutations, although similar, demonstrated specific differences when present in the soluble or membrane-bound context. Our results demonstrate that the electrostatic and flexibility properties of the hinge segment are critical for electron transfer from CPR to its redox partners.

## Introduction

Monooxygenase enzymes occur in all kingdoms of life and cytochromes P450 (P450s) represent the largest superfamily of them (Lamb and Waterman, 2013; Munro et al., 2013). In mammals, microsomal P450s catalyze the oxidation of a wide variety of essential endogenous and xenobiotic compounds (Coon, 2005; Rendic and Guengerich, 2015) by a two-electron activation of molecular oxygen, whereby one atom of oxygen is inserted into the organic substrate and the other is reduced to water (Guengerich, 2007). The catalytic cycle of P450 enzymes depends on redox partners for electron delivery. The NADPH-cytochrome P450 reductase (CPR) is strictly required for the activity of microsomal P450s, while cytochrome *b*_5_ can potentially be a donor molecule, albeit only for the second electron transfer step (Scott et al., 2016).

Human CPR, encoded by the *POR* gene, is a 78-kDa multidomain diflavin reductase that binds both FMN and FAD and is attached to the cytoplasmic side of the endoplasmic reticulum via a transmembrane segment at its N-terminus. The domains that bear the two cofactors are: a flavodoxin-like FMN-binding domain and a ferredoxin-NADP^+^ reductase like FAD-binding domain. The third domain, called the hinge segment, links the FMN and the connecting/FAD domain. Electrons flow through CPR from NADPH to oxidized FAD as a hydride ion transfer to the FAD N5 atom, then, one by one, from the FAD to the FMN, then from the FMN hydroquinone to external acceptors, with the FMN cycling mainly between the hydroquinone and the blue semiquinone states (Murataliev et al., 1999).

Although studies of CPR have predominantly focused on its role in P450 catalysis, CPR also support electron transfer to other enzymes like heme oxygenase (Schacter et al., 1972), squalene epoxidase (Ono and Bloch, 1975), dehydrocholesterol reductase (Nishino and Ishibashi, 2000), and though not uniquely, cytochrome *b*_5_, involved also in fatty acid desaturation and elongation reactions (Oshino et al., 1971). The determinants of this promiscuity in physiological acceptors are not yet known, however, they might indicate that the mechanisms of recognition of this heterogenic group of redox partners by CPR are probably not highly stringent.

The first X-ray diffraction studies on soluble rat CPR showed a surprising compact structure in which the distance of FAD to FMN was about 4 Å, the two isoalloxazine rings almost coplanar and some parts of the FMN domain tightly packed against the rest of the CPR protein (Wang et al., 1997). This conformation, referred thereafter as part of a locked state, was easily ascribed to the conformation allowing electron transfer from FAD to FMN. Several other X-ray structures of CPR displaying the same spatial organization were subsequently obtained (Lamb et al., 2006; Xia et al., 2011b; McCammon et al., 2016). This conformational state as obtained in crystals, also occurs in solution (Vincent et al., 2012). Furthermore, a CPR mutant in which the FMN domain was linked to the FAD domain via a disulfide bridge was competent for electron transfer to the FMN moiety, ultimately proving that the locked state is fit for the internal flavin electron transfer but is unable to reduce external cytochrome acceptors unless the disulfide bridge is reduced (Xia et al., 2011a). This led to the hypothesis that interdomain motions have to take place to render the FMN domain accessible to electron acceptors.

The crystal structure of a mutant CPR in which the hinge segment was shortened by deletion of four residues (ΔTGEE) shows three different molecules per asymmetric unit. In each of them, the FMN domain is in a different position, more and more distant from the rest of the protein, whereas their connecting and FAD-binding domains are strictly superimposable, demonstrating a marked reorientation of the FMN domain relative to the FAD domain (Hamdane et al., 2009). Another evidence for domain mobility has come from the structural study of a chimeric enzyme consisting of the FMN-binding domain of yeast CPR and the remainder of the molecule, including the hinge segment, from human CPR (Aigrain et al., 2009). This chimeric enzyme, which is active in NADPH reduction of cytochromes *c* and P450, displays a single, well-defined, molecule in the asymmetric unit. In this structure, the FMN domain is no longer making any interface with the connecting and FAD domains. Hence, large domain movements can happen in CPR (∼86 Å distance between the two flavins). Furthermore, the redox potential of the flavins exhibit changes that maybe attributed to this large conformational change (Aigrain et al., 2011). The various conformations in which the FMN domain is no longer making an interface with the FAD domain represent an ensemble of structures that can be designated as the unlocked state. SAXS studies on human CPR have also been crucial in demonstrating that, in solution, human CPR is in equilibrium between a closed, compact and a series of open, extended conformations (Ellis et al., 2009; Huang et al., 2013; Frances et al., 2015). The abovementioned mutant CPR containing a disulfide bond, though capable of NADPH-mediated ferricyanide reduction, is essentially incapable of supporting P450 activity unless the disulfide bond is reduced (Xia et al., 2011a). The ability of both flavin domains to move relatively back and forth from each other is thus a prerequisite for the FMN domain to interact with its physiological acceptors. However, the structural determinants that guide this conformational transition between open and closed forms, a process demonstrated to be essential for gating the interflavin electron transfer in CPR and thus to its redox partners, are still not fully identified.

It has long been known that electron transfer from CPR to cytochromes P450 displays a strong ionic strength dependency which demonstrated that the electron transfer complex between the CPR and a P450 is, beside hydrophobic interactions, strongly based on charge pair interactions (Tamburini and Schenkman, 1986; Nadler and Strobel, 1988, 1991; Davydov et al., 1996, 2000; Bridges et al., 1998). Most of these studies evidenced that ionic interactions between CPR and its redox partners were the major determinants of the salt effects on P450s activities and are reviewed in (Hlavica et al., 2003). However, we also demonstrated that the ionic strength effect on cytochrome *c* reduction by the soluble form of human CPR depends on the conformational equilibrium between the locked and unlocked states (Frances et al., 2015). From the comparative studies of the open conformation seen in the chimeric yeast-human CPR, two residues in the hinge, G240 and S243, were proposed to be important molecular determinants for the large conformational changes (Aigrain et al., 2009). Beside these two residues, I245 and R246 were also recently identified, in a molecular dynamics simulation study, as two essential residues of the hinge segment, forming a part of the conformational axis and involved in a rotational movement of the FMN domain relative to the rest of the protein (Sündermann and Oostenbrink, 2013). S243 and R246 were also found to display large chemical shifts during the change in the conformational equilibrium between the locked and unlocked states (Frances et al., 2015). Additionally, the hinge segment was also found to be directly controlling electron transfer to cytochrome *c* from the reductase domain of nNOS (Haque et al., 2007, 2012) as well as in CPR (Grunau et al., 2007).

In this work we have targeted four specific residues of the hinge segment indicated above, for site-directed mutagenesis to test their potential role in the conformational equilibrium of human CPR. By analyzing the salt-dependent changes of the cytochrome *c* reductase activity in the context of both soluble and membrane-bound forms of CPR, we propose several hypotheses on the role played by these residues on the transition between the locked to the unlocked state, and thus in the electron transfer mechanism of CPR.

## Materials and Methods

### Reagents

Dichlorophenolindophenol (DCPIP) and potassium ferricyanide were from Fluka (Buchs, Switzerland). L-Arginine, ampicillin, kanamycin sulfate, chloramphenicol, cytochrome *c* (horse heart), isopropyl β-D-1-thiogalactopyranoside (dioxane-free), thiamine, glucose 6-phosphate, glucose 6-phosphate dehydrogenase, NADP^+^ and NADPH were obtained from Sigma–Aldrich (St. Louis, MO, United States). Phenylmethanesulfonyl fluoride was purchased from Gerbu Biotechnik GmbH (Heidelberg, Germany). Bacto agar, Bacto peptone and Bacto tryptone were obtained from BD Biosciences (San Jose, CA, United States). Bacto yeast extract was obtained from Formedium (Norwich, England). A polyclonal antibody from rabbit serum raised against recombinant human CPR obtained from Genetex (Irvine, CA, United States) was used for immunodetection of the membrane-bound CPR, while the respective antibody used the in the case of the soluble form of the enzyme was obtained from Thermofisher Scientific (Waltham, MA, United States).

### Construction and Cloning of the Different Mutants

#### Soluble Forms of Human CPR

Soluble CPR mutants, deleted of their first 44 N-terminal amino acids, were cloned by the Gibson method using a synthetic DNA fragment containing the desired mutation, with a compatible divergent PCR amplification of the plasmid pET15b expressing the soluble form of the human CPR, containing a N-terminal 6xHis tag. DNA sequencing confirmed the absence of any undesired mutations. The constructed plasmids were transformed into competent *Escherichia coli* BL21 (DE3) cells for expression.

#### Membrane-Bound Forms of Human CPR

Plasmid pLCM_POR (Kranendonk et al., 2008) was used for the expression of the membrane-bound, full-length forms of human CPR. Mutants containing alanine substitutions of residues I245 and R246 were initially obtained through standard site-directed mutagenesis, using the sub-cloning vector pUC_POR, containing the initial segment (1–1269 bp) of human POR cDNA comprising the FMN and hinge domains (based on National Center for Biotechnology Information sequence NM_000941, encoding the CPR consensus protein sequence (NP_000932)). The mutated segments were obtained from the different mutated pUC_POR plasmids, using *Eco*RI + *Aat*II restriction enzymes and cloned back into full-length CPR expression vector pLCM_POR. CPR mutants containing proline substitutions of residues G240, S243, I245 and R246 were obtained by the Gibson method using a synthetic DNA fragment containing the desired mutation and a compatible divergent PCR amplification of the plasmid pET15b expressing the soluble form of human CPR. The mutated gene fragments were sub-cloned in vector pUC_POR using Megaprimer PCR of whole plasmid (MegaWhop) (Miyazaki and Takenouchi, 2002), followed by *Dpn*I treatment. The mutated segments were obtained using *Eco*81I + *Sac*I restriction enzymes and cloned back into the CPR expression vector pLCM_POR. The different constructed pLCM_POR plasmids were transformed into DH5α *E. coli* cells for plasmid propagation. POR cDNA of plasmids was fully sequenced to confirm the introductions of the designed mutations and the absence of any undesired mutations. CPR mutants were expressed in *E. coli* BTC, using the specialized bi-plasmid system for co-expression of CPR with human CYPs (Duarte et al., 2005). The pLCM_POR and the CYP-void plasmid pCWΔ (Kranendonk et al., 2008) were transfected through standard electroporation procedures.

### Protein Expression and Isolation

#### Soluble Forms of CPR

Protein expression in BL21 (DE3) cells was carried out at 29°C in Terrific Broth medium complemented with 1 mg.l^-1^ riboflavin and 100 μg.ml^-1^ ampicillin during 36 h (Frances et al., 2015). Cultures were spun down for 10 min at 7,200 g and suspended in 20 mM Na/K phosphate buffer pH 7.4 (buffer A). Cell lysis was achieved by 4 cycles of sonication (30 s of burst followed by intervals of 2 min for cooling) in buffer A containing a protease inhibitor cocktail (aprotinin 0.3 μM, leupeptin 1 μM, pepstatin A 1.5 μM, benzamidine 100 μM, metabisulfite 100 μM). Cell debris were removed by centrifugation at 8,200 *g* for 1 h at 4°C. His-tagged proteins were bound on a TALON polyhistidine-TAG Purification Resin (Clontech, Mountain View, CA, United States), equilibrated with buffer A, containing 0.25 M NaCl and 0.25 M KCl, washed with this equilibrium buffer and eluted with a 0.25 M imidazole step gradient. Imidazole was eliminated from the eluted fraction by sequential washes in buffer A using a Vivaspin-15 centrifugal concentrator (Sartorius, Goettingen, Germany). The protein solution was stored at 4°C in buffer A, containing 1 μM of both FMN and FAD. Purity of the sample was determined by SDS–PAGE and examination of the 280/450 nm ratio measured by optical spectroscopy.

#### Membrane-Bound Forms of CPR

Expression of the full-length membrane bound CPR mutants was obtained in BTC bacteria and membrane fractions of the different strains were prepared and characterized for protein content as described previously (Marohnic et al., 2010; Palma et al., 2013). CPR content of membrane fractions was quantified by immunodetection against a standard curve of purified human, full-length WT CPR, using polyclonal rabbit anti-CPR primary antibody and biotin-goat anti-rabbit antibody in combination with the fluorescent streptavidin conjugate (WesternDot 625 Western Blot Kit; Invitrogen). Densitometry of CPR signals was performed using LabWorks 4.6 software (UVP, Cambridge, United Kingdom).

### DCPIP and Ferricyanide Reduction Assay and Cytochrome *c* Reduction Microplate Assay

#### DCPIP and Ferricyanide Reduction

With DCPIP, assays were performed in 20 mM Tris-HCl buffer containing 1 mM EDTA, and various NaCl concentrations (from 50 mM up to 1.4 M), pH 7.4 at 25°C in the presence of 70 μM DCPIP. Initial rates were monitored at 600 nm using Δε_M_ = 21,000 M^-1^.cm^-1^. With ferricyanide, assays were performed in the same conditions as described above, but in the presence of 1 mM ferricyanide. Initial rates were monitored at 420 nm using Δε_M_ = 1,020 M^-1^cm^-1^.

#### Cytochrome *c* Reduction Activities

Initial experiments were performed with soluble CPR. Cytochrome *c* reductase activity was followed at 550 nm (Δε_M_ = 21,000 M^-1^.cm^-1^) with CPR concentrations ranging from 2 to 20 nM. In cuvette assays, cytochrome *c* (100 μM final) and NADPH (200 μM final) were mixed in 20 mM Tris-HCl buffer containing 1 mM EDTA, and various NaCl concentrations (from 50 mM up to 1.4 M), pH 7.4 at 25°C. The reaction was initiated by adding CPR. A set of preliminary rate assays were performed in a microtiter plate format to optimize linearity of the reaction traces for the mutants, with conditions close to the traditional cuvette approach. In particular, the concentrations of CPR, cytochrome *c* and NADPH were varied in several control experiments to ensure linearity. In these assays, CPR and NADPH (200 μM final) were mixed and the reaction was started by diluting directly this mix in microplate wells containing 20 mM Tris-HCl buffer pH 7.4, supplemented with cytochrome *c* (100 or 200 μM) and the *ad hoc* NaCl final concentration (from 50 mM to 1.25 M) at 37°C. The reaction was monitored at 550 nm for 5 min in a PowerWave X select microplate reader (Biotek Instruments, Winooski, VT, United States).

A first set of kinetic measurements was performed in triplicate using the two cytochrome *c* concentrations indicated above. A second set of triplicate experiments was repeated with another batch of purified CPR. Reduction velocities were found virtually equal when using cytochrome *c* at 100 or 200 μM (data not shown). Cytochrome *c* was subsequently used in the microplate format at 200 μM (final concentration) for all kinetic measurements. CPR samples (soluble forms) were diluted up to concentrations giving linear velocity traces to compensate for lower or increased velocities, see Supplementary Table S1. Each k_obs_ value is the average of six measurements: one triplicate using a first batch of enzyme and a second triplicate, using a second batch of purified CPR.

The conditions of the microplate rate assay were then verified for the membrane-bound CPRs, to ensure linearity of the reaction traces. Reactions were followed for 4 min in a multi-mode microtiter plate reader (SpectraMax^®^i3x, Molecular Devices) and each sample was assayed at least in triplicate. The optimal dilution of the membrane bound WT CPR was determined (0.5 pmol/ml), using 200 μM of cytochrome *c*, NADPH regenerating system (NADPH 200 μM, glucose 6-phosphate 500 μM and glucose 6-phosphate dehydrogenase 0.04 U/mL, final concentrations) and 372 mM NaCl in a 100 mM Tris buffer (pH 7.4). The same CPR dilution was applied in the cuvette assay under the same conditions except for cytochrome c (50 μM) and NADPH (200 μM, without regeneration) and was measured during 60 s, which resulted in virtually the same k_obs_ as with the microtiter plate format assay. Finally, the membrane bound mutants were assayed with CPR well-concentrations, proportionally diluted as those determined for the soluble forms (see Supplementary Table S1). Control experiments with *E. coli* (BTC) membranes without CPR expression demonstrated no cytochrome *c* reduction upon dilution.

## Results

### Ionic Strength Dependency of the Electron Transfer of the Soluble Form of Human CPR to Three Artificial Acceptors

**FIGURE 1:**
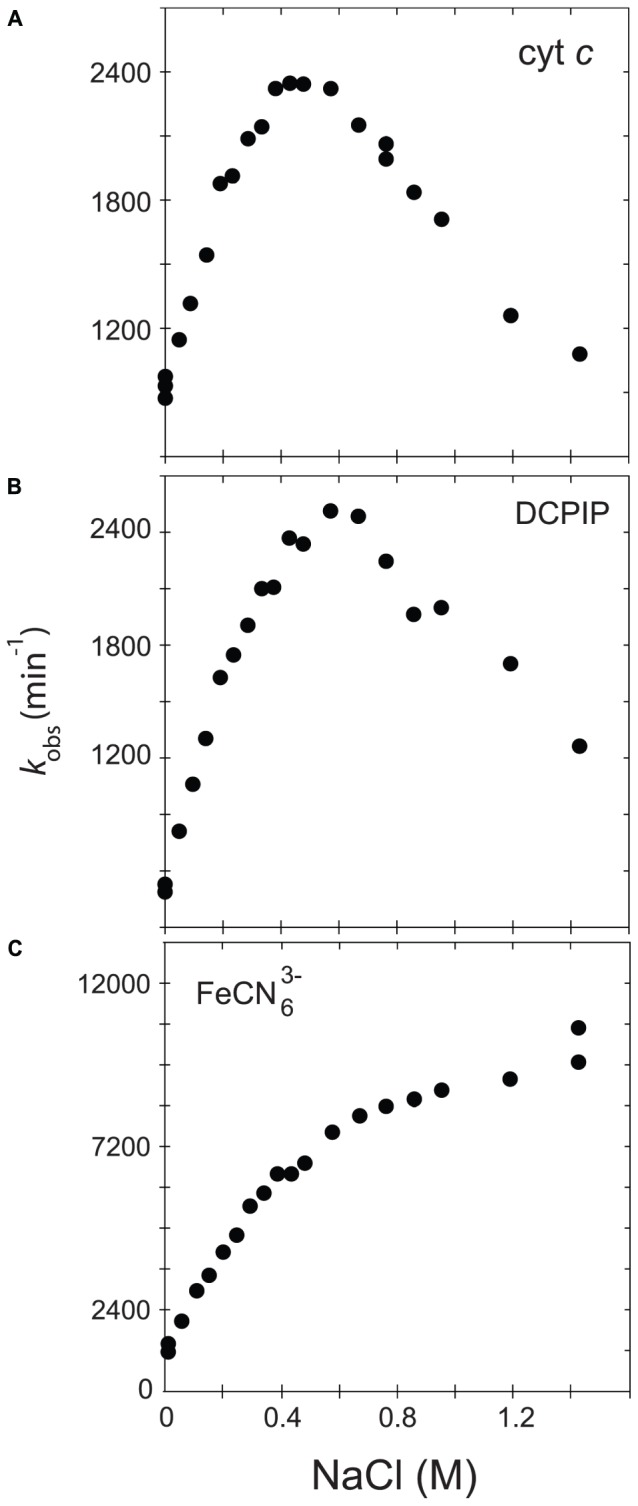
Variation of *k*_obs_ in function of the ionic strength. The activity of the soluble, WT form of human CPR was assayed with various acceptors, namely cytochrome *c* **(A)**, DCPIP **(B)** or ferricyanide **(C)**.

In order to further understand the role of the ionic strength in electron transfer from CPR to acceptors such as cytochrome *c* and discriminate between the two major hypotheses (salt-dependent conformational equilibrium or electrostatic interactions with the substrates), we analyzed and compared the ionic strength dependency of CPR toward DCPIP and ferricyanide with that of cytochrome *c*. DCPIP and ferricyanide have unique characteristics in term of charge and electron receiving capabilities (redox potential). **Figure 1** shows the activity profiles of the soluble, WT form of human CPR in function of the ionic strength for cytochrome *c*-, DCPIP- and ferricyanide-reduction.

Cytochrome *c* and DCPIP related graphs share the same bell-shaped curve. For cytochrome *c*, the concentration of NaCl giving the maximal *k*_obs_ is around 400 mM, a value quite comparable to the one measured in our previous study (Frances et al., 2015). However, the maximum of *k*_obs_ is different for DCPIP (575 mM). This difference might be attributed to the fact that DCPIP is an obligate two electrons (hydride) acceptor, which may have a different overall electron transfer scheme compared to cytochrome *c*. The ionic strength dependent variation of *k*_obs_ measured for the reduction of DCPIP and cytochrome *c* by soluble human CPR display both a bell-shaped profile. However, these substrates are different in term of net charge (cytochrome *c* contains + 9.5 charges at pH 7.0 while DCPIP is neutral). This result thus strengthens the hypothesis that the ionic strength dependency of electron flow from CPR to acceptors is mainly determined by the conformational equilibrium between the locked and unlocked states of CPR and only in a minor manner by electrostatic interactions between the FMN domain and the acceptor.

We also analyzed the reduction of ferricyanide, another non-natural substrate. Interestingly, the salt-dependent *k*_obs_ profile is totally different from the ones seen with cytochrome *c* or DCPIP, being composed of two straight lines crossing at a NaCl concentration of around 450 mM. The constant rise of the *k*_obs_ might be attributed to a gradual increase of the redox potential of ferricyanide due to the increase of ionic strength in Tris-based buffers (O’Reilly, 1973). Therefore, the ferricyanide reduction, which mainly occurs at the FAD cofactor (Vermilion et al., 1981), may be relatively independent from the salt concentration and hence from the conformational equilibrium, a result that was previously observed with the ΔTGEE mutant (Hamdane et al., 2009). Interestingly, the inflection occurs around a salt concentration quite similar to the one yielding the maximum *k*_obs_ with cytochrome *c*. However, we do not have any coherent explanation for the peculiar salt profile observed with this substrate.

Based on these latter results, we decided to use cytochrome *c* reduction as an appropriate reporter to probe salt-mediated conformational equilibrium changes in electron transfer of specific CPR mutants.

### Design of the Different Mutants

The hinge segment is defined by a stretch of 14–15 amino acids that does not display any particularly defined secondary structure. It is also the only part of the CPR structure where evident structural changes can be seen when comparing the two open conformations of CPR (crystallographic structures of the ΔTGEE mutant and the yeast/human chimera) and the other closed conformations of CPR (yeast, human, rat). Residues G240 and S243 (numbering according to the human CPR consensus amino acid sequence NP_000932) correspond to positions with strongly modified phi and psi angles between the closed and open forms (Aigrain et al., 2009). These two residues were therefore mutated into prolines in order to test if and how geometrical constraints modify the conformational equilibrium of CPR. Residues I245 and R246 were selected based on the observation that the backbone atoms of both residues were rotated in the ΔTGEE mutant or in the closed to open transition visualized by molecular modeling (Hamdane et al., 2009; Sündermann and Oostenbrink, 2013). For this last residue, we evidently switched the cationic Arg residue into a non-charged one (either Ala or Pro) while for the previous Ile residue, we preferred mutations affecting either the mobility/rigidity (Ile to Ala or Pro). Two double mutants, cumulating charge and flexibility changes were also designed for residues 245/246. Finally, as the physiological form of CPR is membrane-bound and the membrane anchoring segment may have a profound influence on the equilibrium between locked and unlocked states or the open-closed exchange rates, we analyzed the effects of these mutations in both the soluble and membrane bound context.

**FIGURE 2:**
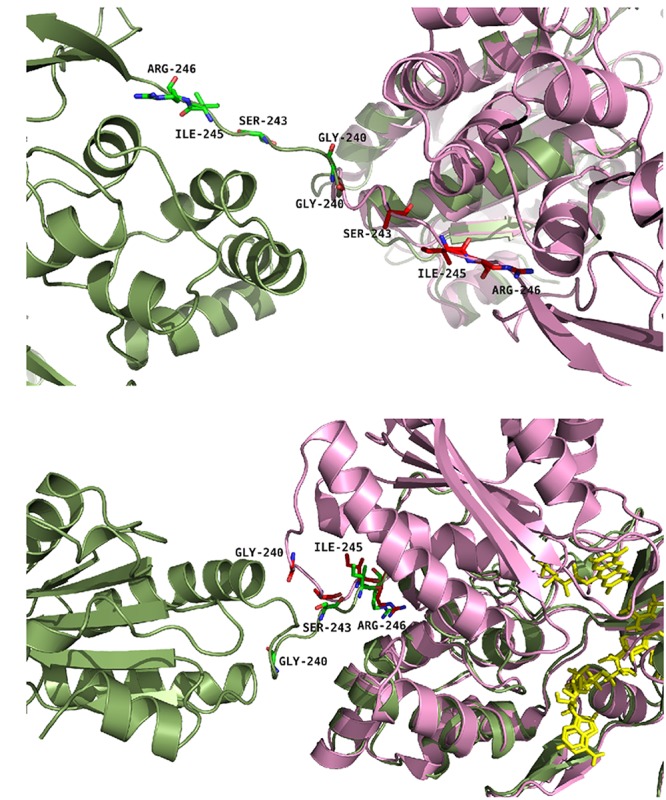
Positions of the studied mutations in CPR. The structure of the yeast-human open form CPR chimera (3FJO, in dark green) and the WT soluble human closed form CPR (5FA6, chain A, in pink) were used to analyze the variations in the hinge segment between the closed and open form of CPR. Mutated residues are displayed as sticks of either green or magenta colors according to the structures. **(A)** Both structures were aligned onto their FMN domains. **(B)** Both structures were aligned onto their connecting/FAD domains. The figure was prepared using the PyMol software (Schrödinger, 2015).

Based on these rationales, six single mutants (G240P, S243P, I245A, I245P, R246A and R246P) and two double mutants (I245A/R246A and I245R/R246I) were produced both in the soluble and membrane-bound (full-length) forms of CPR. **Figure 2** provides a representation of the positions of the four targeted amino acids in the structure of the closed conformation of human CPR (5FA6, chain A) and on the structure of the open conformation of the yeast/human chimeric CPR (3FJO). The two structures (5FA6, chain A and 3FJO) were aligned onto the FAD domain (**Figure 2A**) or the FMN domain (**Figure 2B**). Both views provide the rationale for the design of our mutants: G240 corresponds to the position where the hinge is tilted in the open conformation compared to the closed conformation (**Figure 2A**); S243 side chain orientation is different between the two forms (**Figure 2A**); I245 and R246 show an interesting proximity and potential interaction with the connecting domain (**Figures 2A,B**).

### Production of the Various Mutants of Human CPR

#### Soluble Forms

Eight different plasmids encoding the soluble CPR mutants G240P, S243P, I245A, I245P, R246A, R246P, I245A/R246A and I245R/R246I were introduced into *E. coli* and the various proteins expressed and purified as described in Section “Material and Methods.” SDS-PAGE gel analysis shows that the different mutant proteins present the same profile than the wild type, with a major band just below 70 kDa which corresponds to the soluble form of the CPR (69 kDa). Biological duplication of the entire expression and purification stages was performed to ensure repeatability. Some discrete bands, at lower molecular weights compared to WT CPR were detected either with Coomassie staining or by western blot analysis (see Supplementary Figure S1), indicating some minor degradation of the purified enzymes. However, these represent less than 5% of the full-length soluble CPR and hence would have a minor impact on the kinetic assays. All CPR mutants presented the same absorbance spectra as the WT soluble enzyme, with a maximum of absorption at 450 nm, indicating no or minor changes in the chemical surrounding of both flavins. Last, all mutants were obtained in the semiquinone form, like the WT CPR, indicating no major changes in the absolute redox potential values of the flavins in the mutant forms. These results emphasize that the introduced mutations did not apparently modify the surroundings of the flavins.

#### Membrane-Bound Forms

The eight different mutants were introduced in the *E. coli* BTC strain, expressed and bacterial microsomes were isolated, as described previously (Kranendonk et al., 2008). No expression problems were encountered, except for mutant G240P for which the level of expression was substantially lower than for the other mutants. Immunodetection of CPR in isolated microsomes showed only trace amounts of the G240P mutant protein, not sufficient for subsequent analysis. Other CPR mutants demonstrated expression levels comparable with the WT form (see Supplementary Figure S1).

### Mutations Affect Differently the Soluble and Membrane-Bound Forms of CPR

#### Assay Conditions

**FIGURE 3:**
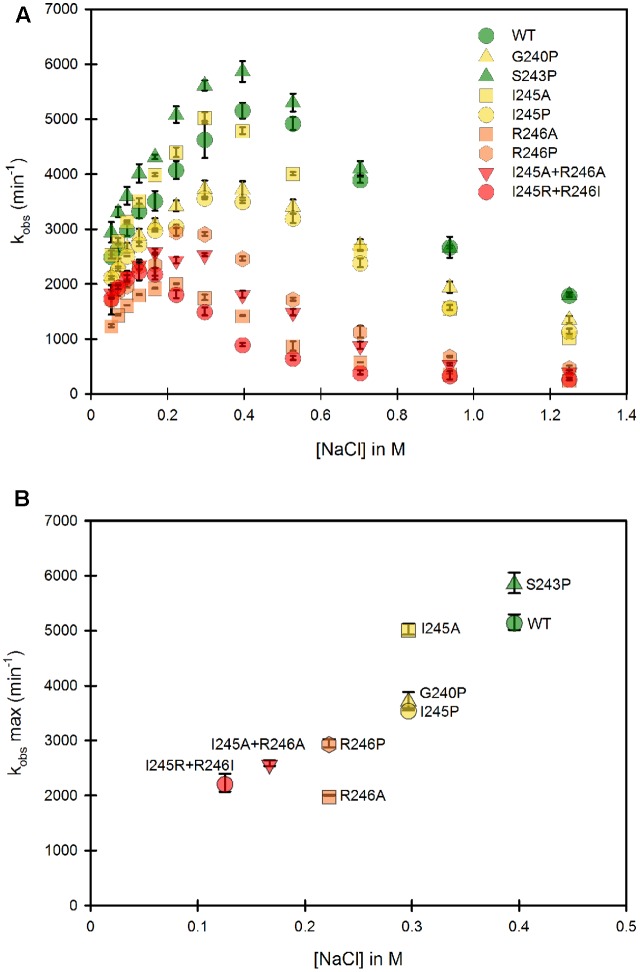
Variation of the *k*_obs_ for cytochrome *c* reduction in function of the ionic strength for the WT and mutants of the soluble forms of human CPR. Cytochrome *c* reduction assays were performed in triplicate in 96 well microtiter plates as described in the Material and Methods section. **(A)** Salt dependent profile of the *k*_obs_ with the WT and the mutants. Symbols depicting the different mutants are included in the panel. **(B)** Maximal *k*_obs_ values were extracted from panel A and plotted as a function of the salt concentration. Symbols depicting the different mutants are identical as the ones in **(A)**. *k*_obs_ values represent the average of three replicates and the error bars depict the standard deviation.

Our study was designed to test the variation of cytochrome *c* reduction in various salt concentration conditions for nine CPR enzymes variants (WT + eight mutants) in the context of the soluble or membrane bound forms. We therefore developed and optimized a 96-well plate format assay to follow cytochrome *c* reduction. These experiments were performed at 37°C, contrarily to the primary test realized at room temperature in cuvette with the WT, soluble form of human CPR (**Figure 1**). Optimal conditions of the microplate rate assay were determined to ensure the linearity of the reaction traces. Initially, assays were performed at two cytochrome c concentrations (100 and 200 μM) in order to ensure that saturation was reached at all salt concentrations, which virtually gave the same velocities (data not shown). Subsequently cytochrome *c* was used at 200 μM. CPR concentrations in the tests were also optimized to obtain linear traces for each mutant (both for the soluble and membrane bound forms) in order to compensate for lower or higher activities (see Supplementary Table S1). We verified that salt profiles were equivalent between the tests performed in the cuvette and in the 96-well plate format (data not shown). **Figure 3A** displays the salt profile of the WT as well as mutants in the context of their soluble form at 37°C. Clearly the WT profile is superimposable to the one in **Figure 1**, although minor differences can be seen, notably for the concentration of salt that gives the maximal value of *k*_obs_ (425 mM at 25°C instead of 395 mM at 37°C). This minor variation could be explained by the temperature shift from 25°C to 37°C, probably favoring the opening mechanism and therefore the unlocked state. Apart from this difference, no other changes were noted and the comparison of the salt profiles of the mutants either in their soluble or membrane bound forms was pursued at 37°C, with the 96 well plate assay format.

#### The Hinge Partly Controls the Conformational Equilibrium of the Soluble CPR Form

As mentioned earlier, the ionic strength dependency of cytochrome *c* reduction by CPR can be partly considered as a direct measure of the proportion of the locked *vs*. unlocked states (Frances et al., 2015) and the bell shaped curve of this ionic strength dependence has been also reported in the electron transfer from the flavodoxin of *Desulfovibrio vulgaris* to cytochrome b_553_ (Sadeghi et al., 2000). We therefore performed a full analysis of the ionic strength dependency of cytochrome *c* reduction for all single and double mutants (**Figure 3**). **Figure 3A** depicts the 8 different ionic strength profiles obtained with the various mutants compared with the WT one. All curves of the *k*_obs_ vs. ionic strength display the same typical bell shaped curve seen with the WT CPR. With the exception of the S243P mutant, all ionic strength profiles are shifted to the left (lower NaCl concentrations). Moreover, nearly all of them (except S243P and I245A) have lower maximal *k*_obs_ values. This result indicates that the various introduced mutations have pronounced effects either on the conformational equilibrium or the electron transfer efficiency, yet all of them are still active (lowest *k*_obs_ is 30% of the one observed with WT CPR). The redox potentials of both flavins and cytochrome *c* are known to depend to some extent on the ionic strength. While for cytochrome *c* these changes are known and relatively small (Gopal et al., 1988), it is difficult to predict the changes that would occur for the flavins. Based on our observation that the microenvironment of flavins is probably not significantly altered between mutants and WT CPR (equal absorption spectra) we have based our working hypotheses on the assumption that the redox potential of the flavins are not functionally different between the mutants and the WT soluble or membrane-bound forms of CPR.

From **Figure 3A**, maximal *k*_obs_ values were extracted and used to generate a graph comparing the salt concentration at which the maximum *k*_obs_ occur and the maximal *k*_obs_ value itself (**Figure 3B**). The case of S243P and I245A is interesting. Both have approximately the same *k*_obs_ (slightly higher than the WT for S243P). However, I245A denotes a clear difference in term of optimal ionic strength contrarily to S243P and WT. Another example is the situation for I245A, G240P and I245P, for which the optimal ionic strengths are identical yet the three mutants have different maximal *k*_obs_ values. Hence these data seem to indicate a separation between the efficiency of cytochrome *c* reduction and the conformational equilibrium of CPR (represented by the salt concentration at which the maximal k_obs_ occurs). However, globally, when the profiles are shifted to lower ionic strength the maximal *k*_obs_ values are lower.

The effects on the conformational equilibrium are interesting and merit further analysis. With the exception of S243P, all mutants either favored the unlocked state or destabilized the locked state. The introduction of a proline residue (G240P, I245P and R246P) probably imposes strong constraints on the hinge and may therefore stabilize discrete conformations of the unlocked state. The removal of the positive charge of the arginine residue at position 246 (R246A) may loosen the potential interactions between the hinge and the connecting domain and also destabilize the locked state. It should also be noted that the double mutants present the maximal displacements in term of salt profile and that the charge suppression at position 246 cannot be compensated by the introduction of an arginine at position 245 (see the double mutant I245R/R246I).

Lower maximal *k*_obs_ values are probably associated to some decrease in the rate constants of one or several electron transfer steps. We already demonstrated that conformational exchange occurs at a much faster rate than electron transfer (Frances et al., 2015). Hence, during the course of steady state activity, CPR opens and closes several times before electrons are transferred from FAD to FMN. This also means that the locked state might actually have several closed conformations, not all of them productive for inter-flavin electron transfer, a feature that has already been described (Hay et al., 2010). Therefore, the mutants might disfavor the occurrence of productive conformations in the locked state, hereby reducing the maximal *k*_obs_ value. Hence, although there would not be a direct relationship between conformational equilibrium and maximal *k*_obs_, the effects of some of the mutations on the destabilization of the locked state could also change the rate limiting step.

### The Hinge Controls the Conformational Equilibrium of the Membrane Bound CPR

**FIGURE 4:**
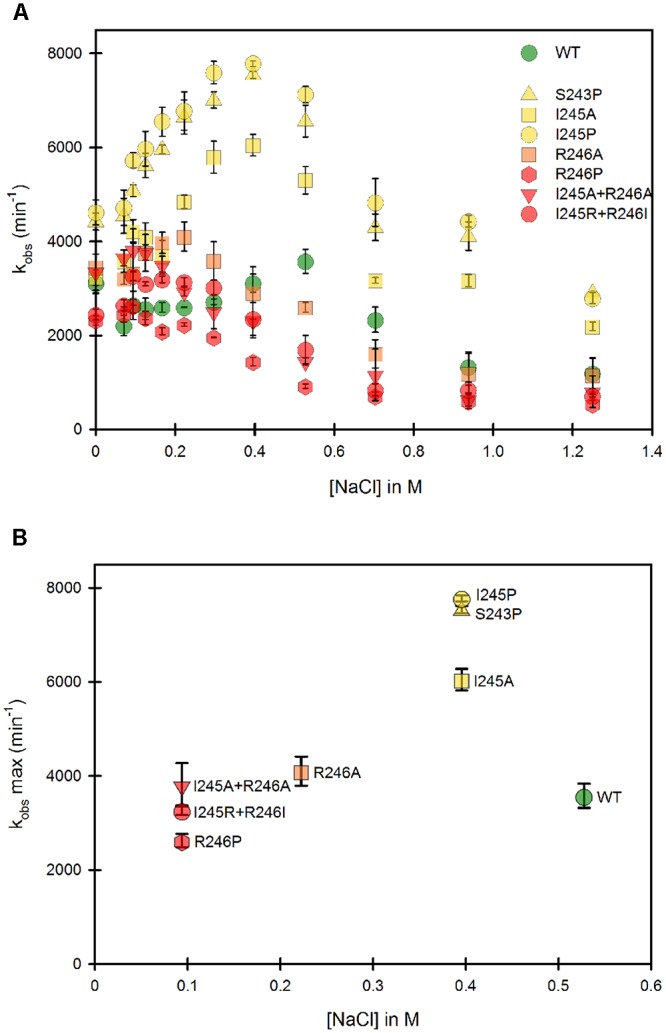
Variation of the *k*_obs_ for cytochrome *c* reduction in function of the ionic strength for the WT and mutants of the membrane bound forms of human CPR. Cytochrome *c* reduction assays were performed in triplicate in 96 well microtiter plates as described in the Material and Methods section. **(A)** Salt dependent profile of the *k*_obs_ with the WT and the mutants. Symbols depicting the different mutants are included in the panel. **(B)** Maximal *k*_obs_ values were extracted from panel A and plotted as a function of the salt concentration. Symbols depicting the different mutants are identical as the ones in **(A)**. *k*_obs_ values represent the average of three replicates and the error bars depict the standard deviation.

The same mutants, when present in the full-length, membrane-anchored form, were subsequently studied. **Figure 4** presents the same analysis of both the variation of *k*_obs_ and the optimal ionic strength value for each of the mutants in the context of the membrane bound form. Our first striking result is that the ionic strength giving the maximum *k*_obs_ for the WT membrane bound form is shifted to higher salt concentrations compared to the WT soluble form, indicating a stronger interaction between the two domains (**Figure 4A**). Various hypotheses can be formulated to account for this fact. First, the presence of the negatively charged phospholipid heads of the membrane probably modifies the electrostatic potential around the FMN domain, potentially strengthening the interactions between the two flavin domains and thus favoring the equilibrium toward the locked state. Second, the presence of the membrane close to the FMN domain might impeach movements of the FAD domain compared to those attainable with the soluble form. This restriction of movement could favor the closing mechanism, hereby stabilizing the locked state.

Despite the above peculiarity of membranous CPR, **Figure 4A** shows that, as seen with the majority of the soluble forms, all mutants have their salt profiles shifted to lower ionic strengths when compared to the WT. This result confirms that the generated mutants have a greater tendency to favor the unlocked state, independently of the presence or absence of the membrane. Although differences exist between individual mutants depending on the context (soluble or membrane bound forms), again the maximal effects are seen with the R246 mutations and the double mutants containing mutations at the positions 246 and 245.

Maximal *k*_obs_ values were also extracted and plotted against the corresponding salt concentration (**Figure 4B**). Overall, the salt concentration at which *k*_obs_ is maximal covers a larger range with the membrane bound forms compared to the soluble forms. This indicates that the influence of salt on the conformational equilibrium is greater when CPR is attached to the membrane than when the various domains have more degree of liberty, i.e., when in the soluble form.

Interestingly, **Figure 4B** shows marked differences in the clustering of mutants compared to **Figure 3B**. This suggests again that the salt concentration giving the highest possible *k*_obs_ (1:1 proportion of locked/unlocked states) does not correlate with the value of the k_obs_ itself. This is particularly evident with the membrane-bound forms of the S243 and I245 mutants.

Some parallels can be drawn between mutants in the soluble and the membrane bound forms from comparison of **Figures 3B**, **4B**: (i) the group which had lower cytochrome *c* reduction rates in the soluble form (R246A, R246P, I245R/R246I and I245A/R246A) have almost the same *k*_obs_ than the WT in the membrane bound form and, (ii) the group that had similar cytochrome *c* reduction rates than the wild type in the soluble form (S243P, I245A and I245P), display a greater efficiency of electron transfer to cytochrome *c* in the membrane bound form.

## Discussion

The role of the hinge segment in diflavin reductase enzymes was studied both in NOS enzymes (Haque et al., 2007, 2012) and in CPR (Hamdane et al., 2009), using either lengthening or shortening of the primary sequence. Our study was designed to address the role of specific residues of the hinge segment of CPR (G240, S243, I245 and R246) without modifying its length. We have analyzed the salt-induced changes in the conformational equilibrium between the locked and unlocked states, via cytochrome *c* reduction, in the context of both the soluble and membrane-bound forms of CPR. In our first set of experiments using the soluble form of WT CPR, we compared the ionic strength profiles of the *k*_obs_ with three artificial substrates. Our results confirmed that the well-known salt dependency of either cytochrome *c* or P450 reductions (Voznesensky and Schenkman, 1992) can be mostly attributed to a change in the conformational equilibrium of CPR, between the locked state, non-competent in electron transfer to cytochrome *c* and the unlocked state capable of transferring electrons from the reduced FMN to acceptors. However, as explained in Section “The Hinge Partly Controls the Conformational Equilibrium of the Soluble CPR Form,” we cannot rule out that the redox potentials of the flavins in the mutants forms of CPR are equivalent. Still, as mentioned before, the fact that the absorption spectra of the different WT and mutant CPRs are indistinguishable indicates that the chemical environment of the flavins is similar in all studied proteins. In the rest of the discussion, we therefore assumed that these redox potentials are equal between all CPR forms studied, bearing in mind that the various properties of the mutants (especially the rate constants) might, to some extent, be attributed to these putative redox potentials changes.

In the membrane bound form of WT CPR, the *k*_obs_ is lower than the one measured with the soluble form, a feature already seen upon solubilization of CPR (Phillips and Langdon, 1962). A possible explanation is a potential restrictive access of cytochrome *c* to the FMN domain caused by the membrane, giving rise to the observed differences between the effects of mutations when present in the soluble or membrane bound forms. Interestingly, none of the introduced mutations were capable of completely abolishing the CPR electron transfer to cytochrome *c* but rather displayed several intermediate features, either changing the salt profile or the efficiency of electron transfer, or both. We thus hypothesize that the targeted positions, although important, are only a part of the structural determinants promoting the conformational exchange between the locked and unlocked states. It is also worth noting that the ionic strength profiles obtained with all mutant forms (soluble and membrane-bound, except for the soluble form of the S243P mutant) were shifted to lower salt concentrations compared to that of the WT CPR. This clearly indicates that the majority of changes that were introduced displaced the equilibrium toward the unlocked state. This is not completely surprising for the mutations that potentially enhance the flexibility (I245A) or removed salt bridges between the hinge and the connecting domain (R246A). However, for the mutations introducing proline residues in the hinge (G240P, S243P, I245P, R246P) the shift in the ionic strength profile might indicate that rigidifying these positions could result either in the destabilization of the locked state or the stabilization of the unlocked state. Still, the maintenance of a rather effective cytochrome *c* reduction in all cases might indicate that the loss of flexibility in the mutants bearing a proline residue may also be partly compensated by the hinge flexibility involving residues at other positions.

The second interesting feature of our mutants is that we can separate the ionic strength profile from the efficiency of the electron transfer to cytochrome *c*, i.e., there is no evident direct relationship between the salt concentration at which *k*_obs_ is maximal and the value of *k*_obs_ itself (**Figures 3B**, **4B**). Thus, in our conditions, the rate limiting steps remain independent of the conformational equilibrium. The conformational exchange frequency between the locked and unlocked states is at minimum 600 s^-1^ (Frances et al., 2015), a value 10 times greater than the *k*_obs_ observed with cytochrome *c* and coherent with the one determined by Haque et al. (2014). This also means that the locked state might actually have several closed conformations, not all of them productive for inter-flavin electron transfer, a feature that has already been described (Hay et al., 2010). Hence, there would not be a direct relationship between conformational equilibrium and maximal *k*_obs_ and the effects of our introduced mutations, destabilization of the locked state, could also affect the rate limiting step giving rise to either equal, lower or higher k_obs_ values compared to the WT forms.

In the soluble form of human CPR, only the S243P and possibly the I245A mutations have no effect on the rate of cytochrome *c* reduction. However, in the membrane bound form, these two mutants, along with I245P, present cytochrome *c* reduction rates higher than the WT form, and higher than the soluble counterparts. To our knowledge, this is the first report of *k*_obs_ values up to 8,000 min^-1^ for electron transfer to cytochrome *c* with CPR. However, these features were already observed in some mutants affecting the charge pairing between the FMN and FAD domains of nNOS (Haque et al., 2013) and in a C-terminal-truncated version of murine iNOS in which cytochrome *c* reduction attained 31,467 min^-1^ (Roman et al., 2000). In the nNOS mutants, the increase of *k*_obs_ for cytochrome *c* reduction was attributed to a change of the conformational behavior. However, in our case, the maximal *k*_obs_ was determined at a salt concentration where the conformational equilibrium corresponds to a 1:1 ratio of locked/unlocked states, as we demonstrated before (Frances et al., 2015). Therefore, the high maximal *k*_obs_ values observed in the mutants may be attributed to a change in the FAD to FMN electron transfer rate limiting step, because, in the case of cytochrome *c* reduction by CPR, this step is considered the slowest. Still, it should be reminded here that natural redox partners of CPR such as microsomal P450, may have specific structural requisites to bind CPR and form productive electron-transfer complexes with its membrane anchored redox partners, a situation evidently different with soluble partners such as cytochrome *c*. The differences in our results for the soluble and membrane-bound forms seem to indicate that residues S243 and I245 of human CPR are playing a role in this specific aspect.

**FIGURE 5:**
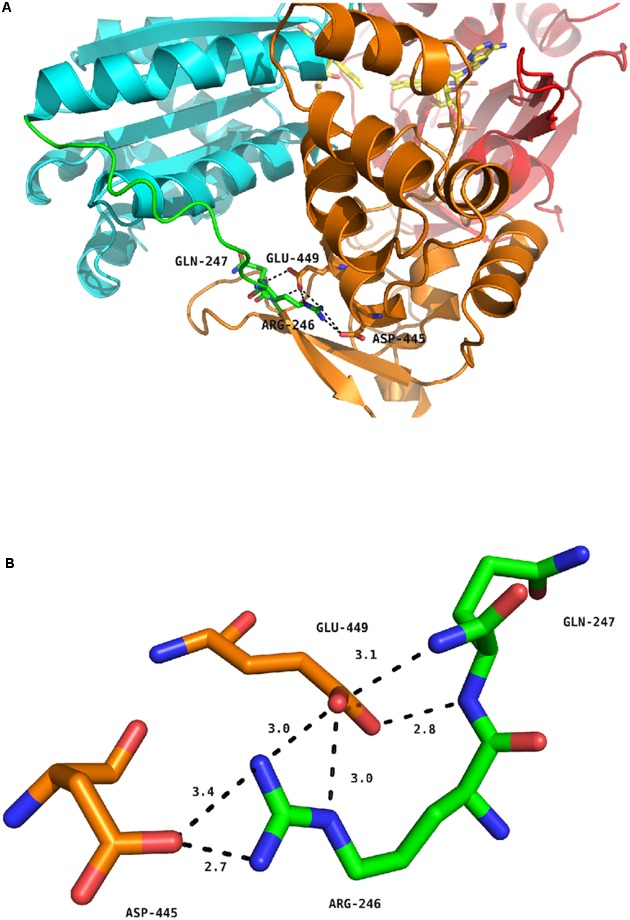
Hydrogen bond network around residue R246 of human CPR. **(A)** Position of R246, Q247, D445 and E449 in the structure of the soluble form of WT human CPR. The color coding is as follows: FMN domain, cyan; hinge segment, green; connecting domain, orange; FAD domain, red. Flavins are depicted in yellow sticks, the various positions as stick in the same color code of the domain to which they belong. **(B)** Detailed view of the hydrogen bonding network. The color coding is the same as in **(A)**.

**Figure 5** depicts the position of the R246 amino acid in the structure of the WT, soluble form of human CPR (McCammon et al., 2016). The R246 is in close contact with two acidic residues (D445 and E449) present in the connecting domain (**Figure 5A**). Mutations at position 246 abolish a charge pairing with these two acidic residues. The distances of the three nitrogen atoms of R246 (NE, NH1 and NH2) with both terminal oxygens of D445 and E449 and the carbonyl oxygen of D445 are compatible with hydrogen bonds (**Figure 5B**). Furthermore, one terminal oxygen atom of E449 is also in a distance compatible to form a hydrogen bond with the amide nitrogen atom of Q247. This relatively strong hydrogen bonding network is evidently disrupted in the mutants changing R246 into any non-charged residue. The addition of a charge at the previous residue (in the double mutant I245R/R246I) does not restore the WT phenotype because I245 side-chain is clearly not oriented favorably in order to substitute for R246 in the complex hydrogen bonding network. This result is reminiscent of the mutations analyzed by Shen and coworkers demonstrating a similar shift in the salt profile when removing potential ionic interactions between the FMN and the connecting domains by mutating E216 into a glutamine residue (Shen and Kasper, 1995). Our results thus demonstrate that the hinge segment also actively participates in the construction of the FMN/FAD interface. When the loss of critical ionic interactions modifies the equilibrium between the locked and unlocked states, the ionic strength at which the maximal value of *k*_obs_ occurs is evidently lower, yet the maximum *k*_obs_ value may remain roughly constant, as demonstrated by several of the mutant CPRs.

A final remark should be made concerning the salt concentration at which the maximal *k*_obs_ was observed for the WT, membrane bound form of CPR, which is around 500 mM NaCl. It is clear that the ionic strength at this salt concentration is much higher than the physiological one, 154 mM KCl (physiological serum). As such it seems that the conformational equilibrium of CPR, *in vivo*, is probably in favor of the locked state, thereby reinforcing the idea of an external trigger for opening CPR (Grunau et al., 2006, 2007), such as the presence of membrane-bound P450s which are physiologically outnumbering CPR molecules by a factor of 5–10 in the liver. How this competition for CPR is affected by the conformational equilibrium is still an open and intriguing question.

## Author Contributions

Experimental design: all authors; experimental work: DC, TL, PU, FE, and SB; data analysis and interpretation: all authors; writing, reviewing and editing of the manuscript: all authors; funding acquisition: GT and MK.

## Conflict of Interest Statement

The authors declare that the research was conducted in the absence of any commercial or financial relationships that could be construed as a potential conflict of interest. The handling Editor is currently co-organizing a Research Topic with one of the authors MK, and confirms the absence of any other collaboration.

## Abbreviations

CPR: NADPH cytochrome P450 reductase
DCPIP: 2,6-dichlorophenolindophenol
FMN: flavin mononucleotide
FAD: flavin adenine dinucleotide
k_obs_: observed rate constant
NOS: nitric oxide synthase
P450: cytochrome P450
WT: wild-type.
