# Correction: The Hinge Segment of Human NADPH-Cytochrome P450 Reductase in Conformational Switching: The Critical Role of Ionic Strength

**DOI:** 10.3389/fphar.2018.00175

**Published:** 2018-03-19

**Authors:** Diana Campelo, Thomas Lautier, Philippe Urban, Francisco Esteves, Sophie Bozonnet, Gilles Truan, Michel Kranendonk

**Affiliations:** ^1^Center for Toxicogenomics and Human Health (ToxOmics), Genetics, Oncology and Human Toxicology, NOVA Medical School, Faculdade de Ciências Médicas, Universidade Nova de Lisboa, Lisboa, Portugal; ^2^LISBP, Université de Toulouse, CNRS, INRA, INSA, Toulouse, France

**Keywords:** diflavin reductase, protein dynamics, multidomain proteins, conformational exchange, electron transfer, protein–protein interaction

There is a mistake in the values of the y axis of Figure 1. The correct version of Figure 1 appears below. The authors apologize for the mistake. This error does not change the scientific conclusions of the article in any way.

**Figure 1:**
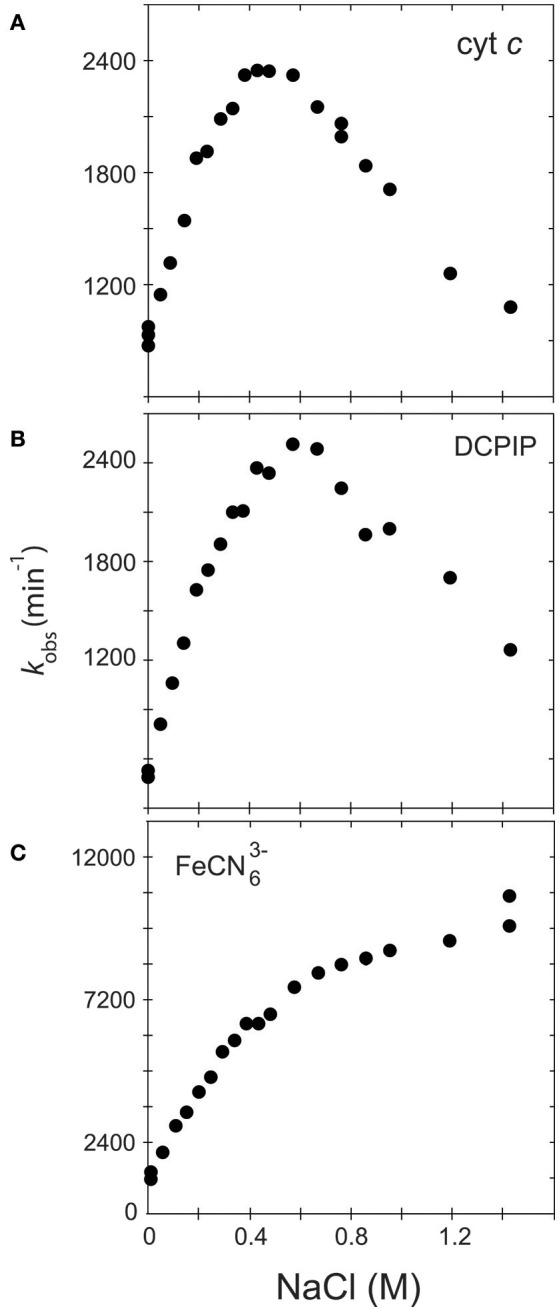
Variation of *k*_obs_ in function of the ionic strength. The activity of the soluble, WT form of human CPR was assayed with various acceptors, namely cytochrome *c* **(A)**, DCPIP **(B)** or ferricyanide **(C)**.

The original article has been updated.

## Conflict of interest statement

The authors declare that the research was conducted in the absence of any commercial or financial relationships that could be construed as a potential conflict of interest.

