# Supplementary material for: The Hinge Segment of Human NADPH-Cytochrome P450 Reductase in Conformational Switching: The Critical Role of Ionic Strength
Source: Front Pharmacol. 2017 Oct 30;8:755. doi: 10.3389/fphar.2017.00755 (PMC5670117; doi:10.3389/fphar.2017.00755)
Supplement: Supplementary file 1 [file Table_1.PDF]

Supplementary Table S1: CPR concentrations used in cytochrome *c* reduction microplate assays.

	Soluble CPR (nM)	Membrane-bound CPR (nM)
WT	6,1	0,50
G240P	11,6	-
S243P	2,8	0,23
I245A	9,2	0,75
I245P	4,4	0,36
R246A	8,8	0,72
R246P	9,4	0,77
I245R+R246I	19,2	1,57
I245A+R246A	4,4	0,36
