# Supplementary figures and images for: The Hinge Segment of Human NADPH-Cytochrome P450 Reductase in Conformational Switching: The Critical Role of Ionic Strength
Source: Front Pharmacol. 2017 Oct 30;8:755. doi: 10.3389/fphar.2017.00755 (PMC5670117; doi:10.3389/fphar.2017.00755)

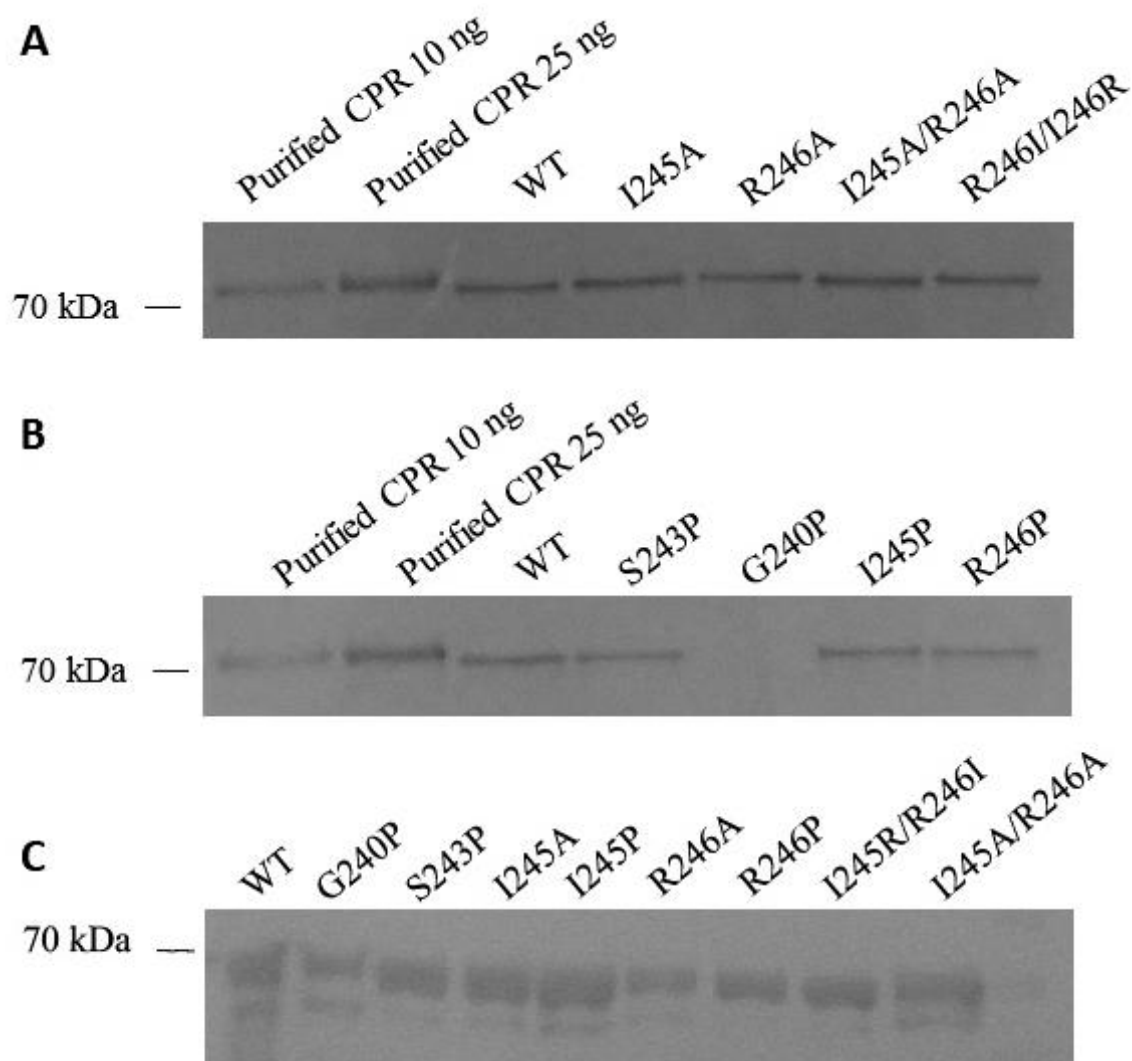

**Supplementary Figure S1.** Immuno-detection of membrane-bound (**A**, **B**) and soluble forms of human CPR (**C**).

Supplement: Supplementary file 2 [file Image_1.PDF]
